# Melioidosis as a Mystique Infection: A Study From Central India

**DOI:** 10.7759/cureus.43439

**Published:** 2023-08-13

**Authors:** Vaibhav Yadav, Akash Pawar, Mahadev Meena, Sagar Khadanga, Ayush Gupta, Tarini Prasad Dandasena, Abhishek Singhai, Rajnish Joshi, Saurabh Saigal, Mahendra Atlani

**Affiliations:** 1 General Internal Medicine, Mahatama Gandhi Memorial Medical College, Indore, Indore, IND; 2 General Medicine, All India Institute of Medical Sciences, Bhopal, Bhopal, IND; 3 Internal Medicine, All India Institute of Medical Sciences, Bhopal, Bhopal, IND; 4 Microbiology, All India Institute of Medical Sciences, Bhopal, Bhopal, IND; 5 Department of Anaesthesiology, All India Institute of Medical Sciences, Bhopal, Bhopal, IND; 6 Nephrology, All India Institute of Medical Sciences, Bhopal, Bhopal, IND

**Keywords:** b. pseudomallei, mystique, arthritis, diabetes mellitus, melioidosis, infectious diseases

## Abstract

Introduction: Melioidosis is caused by the Gram-negative bacilli Burkholderia pseudomallei, which is found in contaminated water and soil and spreads via inhalation, inoculation, and ingestion. Melioidosis manifests diversely in immunocompetent and immunocompromised patients, ranging from asymptomatic to life-threatening respiratory distress, septic shock, localized tissue infection, necrotizing pneumonia, and soft organ abscesses.

Methods: An 18-month observational study was conducted at a tertiary center in central India among various confirmed melioidosis cases, with data gathered and analyzed. Aerobic culture and sensitivity were performed in all studied cases, either in blood/body fluid/localized collection - using blood agar media for the culture and disc diffusion method on Mueller Hinton agar for sensitivity. Other tests, such as radiological imaging, were conducted according to symptoms and signs of localized infection.

Results: The melioidosis cases under study were compared on various clinical/presenting parameters. Melioidosis has a variety of risk factors, but we found that, in India, diabetic patients are at a higher risk of this infection, particularly fatal forms, as all of the patients in our study were diabetic. Melioidosis is known to have joint involvement, either as a source of infection or later in the course of the disease. All cases were successfully treated with antibiotics and surgical procedures, demonstrating the significance of determining disease etiology, early diagnosis, and rapid early management.

Conclusion: Melioidosis is a potentially fatal disease, particularly in diabetics, with a wide range of symptoms and complications. Physicians face a variety of challenges, including clinical symptoms resembling other chronic illnesses, such as tuberculosis, delays in laboratory confirmation, underdiagnosis, reduced reporting, and a lack of suspicion. Because there are very little data and it is a seldom reported infection from central India, we are publishing a study on seven melioidosis patients.

## Introduction

Melioidosis, also known as Whitmore’s disease, is an infectious disease that can infect animals and humans. This disease is caused by Burkholderia pseudomallei (B. pseudomallei), which is a Gram-negative bacillus found in contaminated water and soil [1-3]. It can spread to humans and animals through inoculation, inhalation, or ingestion. The person-to-person mode of transmission is rare, and transmission of melioidosis via sexual contact remains unproven [4]. B. pseudomallei infection can cause a wide spectrum of conditions, ranging from asymptomatic infection to local abscesses, lower respiratory tract infection, and disseminated disease [5].

Melioidosis is a considerable cause of fatal community-acquired pneumonia and septicemia in endemic regions, with mortality rates as high as 44%. Acute pulmonary infection is the most commonly diagnosed form of melioidosis. Pneumonia may be asymptomatic or may present as a severe necrotizing disease [6]. While melioidosis infections have occurred all over the world, Southeast Asia and Northern Australia are the areas where it is most commonly found, with occasional cases in India and China [4]. It has been a less suspected, under-diagnosed, and under-reported disease in the Indian subcontinent [7], with nearly 1,550 out of the total 1,700 cases (more than 90%) reported from India in the last 10 years [8].

Patients with diabetes mellitus are more prone to get melioidosis. The association of melioidosis with diabetes mellitus could be due to the defect in innate immunity of diabetic patients, along with poor glycaemic control. Acute cases of melioidosis with diabetes mellitus showed decreased cellular adaptive immune response as compared to acute melioidosis cases in non-diabetic patients [9]. Bone and joint involvement in melioidosis are rarely reported but are well-established entities. The knee joint is the most commonly affected, followed by the ankle, hip, and shoulder [10]. The sternoclavicular joints can also be involved in the disseminated form of musculoskeletal melioidosis [11].

We present a case series of seven melioidosis cases to emphasize the importance of suspecting, evaluating, and ruling out B. pseudomallei as an etiological agent when any patient presents with a similar symptom profile because our medical facilities are now well-equipped to diagnose it, treat it (more effectively when diagnosed early), and save lives.

## Materials and methods

This case series study was conducted at All India Institute of Medical Sciences, Bhopal, which is a tertiary care center in Central India. The study was performed in the general medicine ward and intensive care unit of the hospital, which had fully equipped procedure rooms and laboratory facilities, with experienced physicians and radiologists.

It was an 18-month observational study conducted among various confirmed melioidosis cases, with data gathered and analyzed. Data regarding chronic illness, occupation, addiction, presentation, and duration of illness were collected and analyzed. A confirmed melioidosis case is defined as one or more clinical samples that are culture-positive for B. pseudomallei. Aerobic culture and sensitivity were performed in all study cases, either in blood/body fluid/localized collection - using blood agar media for the culture and disc diffusion method on Mueller Hinton agar for sensitivity. Antibiotic susceptibility testing has been reported by interpreting the raw MIC of the isolate using vitek 2 BY CLSI-M-45 and CLSI M-100. Other tests, such as radiological imaging, were conducted according to symptoms and signs of localized infection. Blood culture was taken in bottles containing nutrient broth and anticoagulants. Computed tomography was performed by a 128-slice Siemens machine (SBM Healthcare India Private Limited, New Delhi, Delhi) using iodinated contrast. Pre- and post-contrast images were taken and analyzed. Ultrasound-guided liver abscess aspirations were done using aseptic precautions by a radiologist.

All study participants received thorough explanations of the study's objectives, methods, potential advantages, and risks prior to their participation. They received this information in a language they could understand, both verbally and in a written document. The participants were assured that their participation was completely voluntary and would not have any bearing on their medical care if they chose not to participate. They were also given plenty of time to ask questions. They were required to sign the informed consent form after they had read the study carefully, stated their willingness to participate, and understood it completely. Thumbprints were taken in place of signatures for individuals who were unable to sign. Before beginning any data collection or study-specific procedures, all consents were obtained in order to protect participants' rights and autonomy. Throughout the whole study, anonymity and confidentiality were maintained. Microsoft Excel (Microsoft Corporation, Redmond, Washington, USA) was used to enter the information gathered from the study participants' medical records into a computerized database.

## Results

This study includes data from seven melioidosis cases. The clinical symptoms and relevant investigations of which are summarised in Tables 1-2. Case histories and clinical outcomes are also presented below.

**Table 1:** Table showing clinical features and relevant investigations of melioidosis cases (Cases 1-4) CECT- Contrast-enhanced computed tomography; CBNAAT - Cartridge-based nucleic acid amplification test; USG - Use of ultrasonography

Case details	Case 1 (45 years old/male)	Case 2 (48 years old/male)	Case 3 (42 years old/male)	Case 4 (45 years old/male)
Case summary				
Diabetes	Yes	Yes	Yes	Yes
Fever	Yes	Yes	Yes	Yes
Respiratory/abdominal symptoms	No	Yes	Yes	Yes
Weight loss	Yes	Yes	NA	NA
Joint involvement	Yes	Yes	Yes	Yes
Duration of illness	3 months	3 months	15 days	2 months
Significant lab reports				
Hemoglobin	7.2 g/dl	12 g/dl	12.7 g/dl	9.7 g/dl
Total leucocyte count	9210 /dl	4,870 /dl	19,870 /dl	20,530 /dl
Serum albumin	2.75 g/dl	2.67 g/dl	2.06 g/dl	3.8 g/dl
C-reactive protein	248 mg/L	98.5 mg/L	253 mg/L	161 mg/L
Procalcitonin	NA	NA	17 ng/l	NA
Microbiology reports				
Culture (B. pseudomallei)				
Blood	Sterile	Yes	Yes	Sterile
Urine	Sterile	NA	NA	Sterile
Pus/synovial	Yes	NA	Yes	Yes
Sputum	NA	Sterile	NA	NA
Sensitivity (carbapenem)	Yes	Yes	Yes	Yes
CBNAAT (pus/sputum)	Negative	Negative	NA	Negative
Radiological reports				
USG abdomen	Liver abscesses	NA	NA	NA
Chest x-ray	NA	Bilateral lung patchy consolidations	Bilateral lung patchy consolidations	Left lung lower zone consolidation
CECT chest	Right upper lobe lung consolidation with pleural effusion	Lymphadenopathy lung abscesses bilateral effusion	NA	NA
CECT abdomen	Liver & spleen abscess	Hepato-splenomegaly	NA	Liver abscess

**Table 2:** Table showing clinical features and relevant investigations of melioidosis cases (Cases 5-7) CECT- Contrast-enhanced computed tomography; NA- Not available; CBNAAT - Cartridge-based nucleic acid amplification test; USG - Use of ultrasonography

Case details	Case 5 ( 57 years old/male)	Case 6 ( 43 years old/male)	Case 7 ( 32 years old/male)
Diabetes	Yes	Yes	Yes
Fever	Yes	Yes	Yes
Respiratory/abdominal symptoms	Yes	No	Yes
Weight loss	NA	No	Yes
Joint involvement	Yes	Yes	Yes
Duration of illness	15 days	10 days	15 days
Significant lab reports			
Hemoglobin	9.2 g/dl	13.9 g/dl	9.9 g/dl
Total leucocyte count	26,110	10,730	14,590
Serum albumin	2.53 g/dl	3.12 g/dl	2.98 g/dl
Procalcitonin	8.93 ng/l	NA	NA
Microbiology reports			
Culture (B. pseudomallei)			
Blood	Yes	Yes	Sterile
Urine	Sterile	Sterile	Sterile
Pus	NA	NA	Yes
Sputum	NA	NA	NA
Sensitivity to carbapenem	Yes	Yes	Yes
CBNAAT (pus/sputum)	NA	NA	Negative
Radiological reports			
USG abdomen	NA	Grade 2 fatty liver	Multiple liver abscesses
Chest x-ray	Bilateral upper lobes patchy opacities Consolidation	Normal	Normal
CECT abdomen	NA	NA	Multiple small liver abscesses in both lobes, splenic infarct with few splenic abscesses, and pancreatic abscess
CECT chest	NA	No significant abnormalities detected	Focal areas of cavitary consolidation in the right middle lobe with bilateral pleural effusion

Case 1

**Figure 1:**
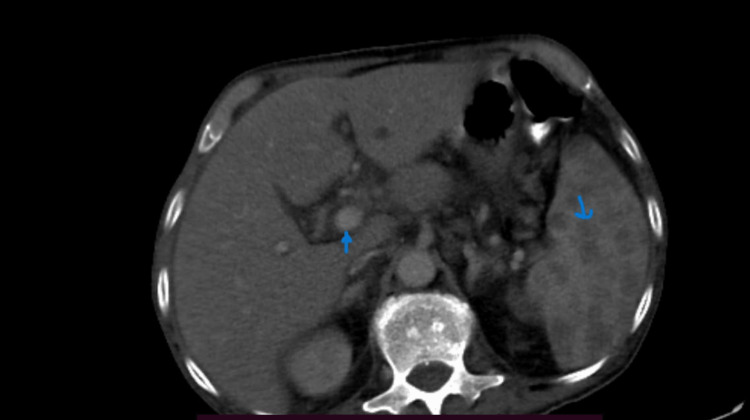
CT image showing multiple multiple liver abscesses and splenic abscesses

A 45-year-old farmer, with recently diagnosed diabetes mellitus, was referred to our center with a complaint of high-grade fever, with chills and rigors for three months, accompanied by significant weight loss. The patient was assessed, and ultrasound of the abdomen revealed several liver abscesses, which were treated with broad-spectrum antibiotics. Blood culture was negative for aerobic and anaerobic organisms. Patient did not respond to treatment. The patient underwent contrast-enhanced computed tomography (CECT), which shows multiple small non-enhancing hypodense areas, arranged in a honeycomb pattern in the liver and splenic parenchyma and patchy consolidation in posterior segments of the right lower lobe (Figure 1). Aspiration of liver lesions revealed pus, and a culture was sent. Disseminated tuberculosis was suspected, but acid-fast staining and the cartridge-based nucleic acid amplification test (CBNAAT) of the pus sample were negative.

Gram stain for pus culture revealed Gram-negative bacilli, culture grew B. pseudomallei, and patient was started on injection meropenem as per senstivity report. During the hospital stay, the patient developed synovitis of both ankle joints, which responded to NSAIDS. Aspiration of the joint fluid revealed an inflammatory picture with 25,000 cells and 85% polymorphs. However, the culture turned out to be sterile. Because the patient also had HLA B-27-positive, reactive arthritis was considered. The patient responded well to carbapenem, the fever subsided, and inflammatory markers were reduced with 14 days of parenteral therapy. The patient was discharged with three months of oral cotrimoxazole and came to follow up with resolving lesions on ultrasonography. 

Case 2

**Figure 2:**
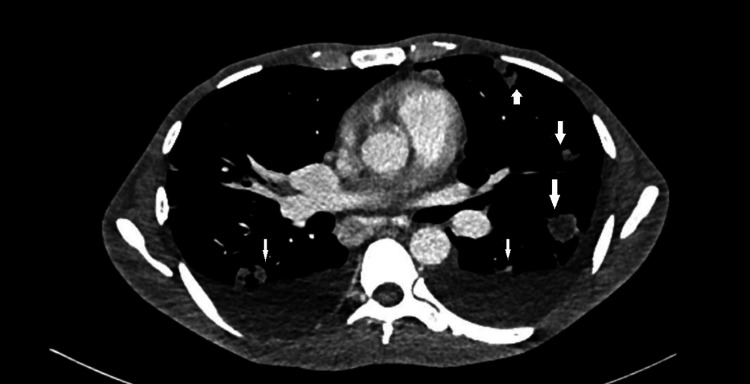
CT chest image showing bilateral small lung abscesses with pleural effusion

A 48-year-old diabetic male, farmer by profession, came to our center with complaints of fever for three months, along with dry cough, significant weight loss, progressive left-side foot drop, and left ankle swelling. Chest X-ray was suggestive of bilateral upper and middle zone patchy consolidation. Sputum for AFB and CBNAAT, along with culture for aerobic organisms, came out to be negative. The patient’s blood culture grew B. pseudomellai. CECT revealed multiple discrete mediastinal lymph nodes, along with multiple small lung abscesses in bilateral lung fields (Figure 2). The patient was started on imipenem as per sensitivity report. After 21 days of parental antibiotic therapy, the patient improved. The fever subsided, and ankle cellulitis improved. Physiotherapy and a splint were done for the foot drop. The patient was discharged with oral minocycline for 30 days.

Case 3 

A 42-year-old male driver, who had recently been diagnosed with diabetes, reported to us with complaints of fever for 15 days, swelling and redness in the right upper and left lower limbs, and shortness of breath for one day. The right upper and left lower limbs have cellulitis, while the left knee has septic arthritis.

**Figure 3:**
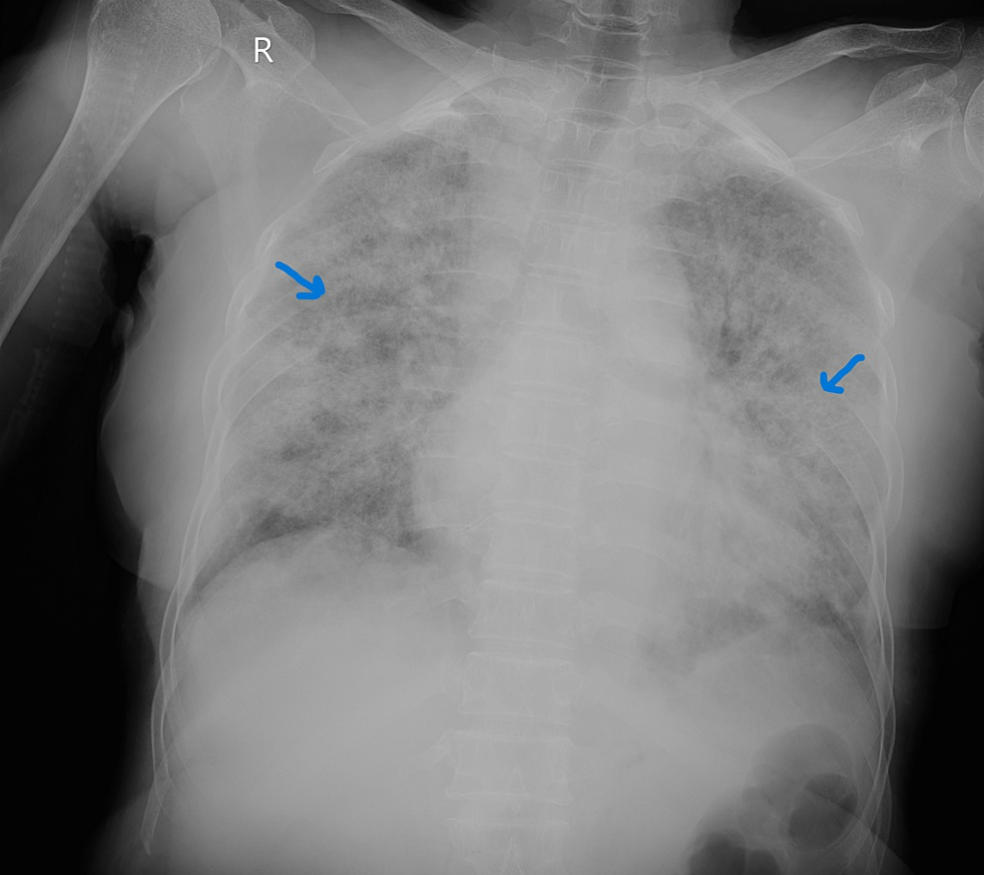
Chest x-ray image showing bilateral patchy lung consolidations

The patients had septic shock, and acute respiratory distress on presentation and managed accordingly on lines of septic shock and put on ventilator support, along with broad-spectrum antibiotics and inotropes. Chest x-ray suggestive of bilateral patchy lung consolidations (Figure 3). Later, the patient underwent left knee arthrotomy and debridement for left knee septic arthritis. Blood culture and left knee aspirate both grew B. pseudomallei susceptible to ceftazidime, imipenem, meropenem, and cotrimoxazole. The patient started on meropenem, and after 10 days of parenteral antibiotic therapy, the patient improved and weaned off from the ventilator.

The patient improved and was discharged after three weeks of therapy of carbapenem. The patient was discharged on oral cotrimoxazole in stable condition.

Case 4

A 45-year-old diabetic male came with complaints of fever with chills and pain in the abdomen for two months. The patient also had a background history of splenectomy for splenic abscess two years back and had taken anti-tubercular drugs for six months.

**Figure 4:**
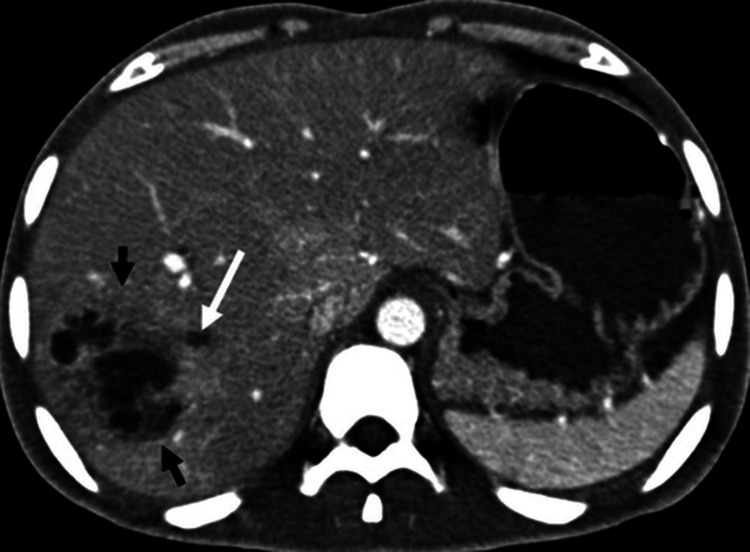
CT image showing multiple round to oval peripherally enhancing hypodense lesions in the liver, suggestive of pyogenic liver abscess

The patient was evaluated, and CECT of the abdomen was done, which shows multiple round to oval peripherally enhancing hypodense lesions in the liver suggestive of pyogenic liver abscess. Ill-defined heterogeneous tissue with adjacent fat stranding was also noted in the left hypochondrium, which is likely residual splenic tissue (Figure 4).

X-ray chest shows left lower zone consolidation. Blood culture, and urine culture came out to be sterile. Aspirate from liver lesions was done which demonstrate pus and culture grew B. pseudomallei which was sensitive to carbapenem, cotrimoxazole and ceftazidime. After 17 days of parenteral meropenam therapy patient was discharged or oral cotrimoxazole for 3 months. On follow up after 6 month there was near complete resolution of liver lesions and chest x-ray. 

Case 5

**Figure 5:**
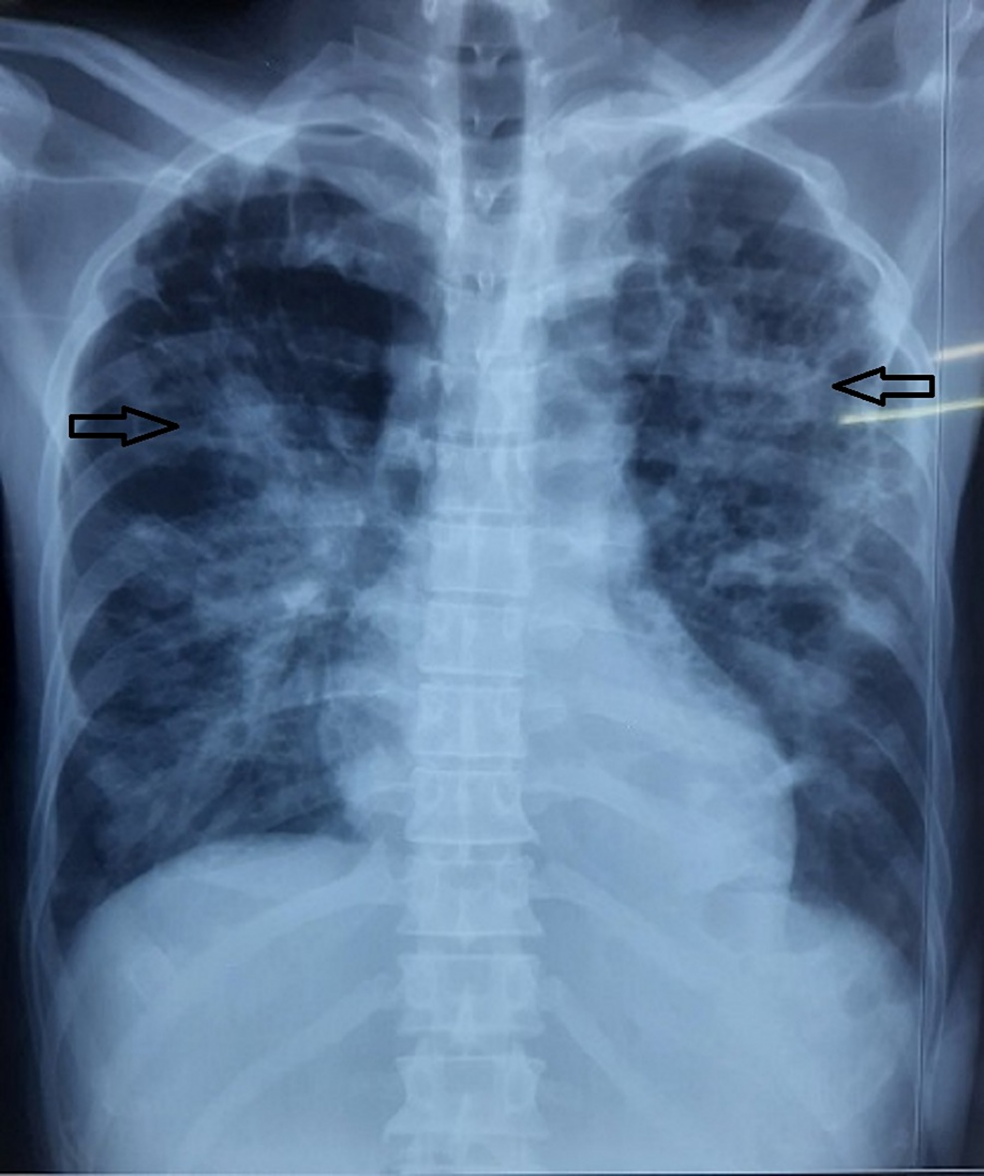
Chest X-ray showing bilateral patchy consolidation

**Figure 6:**
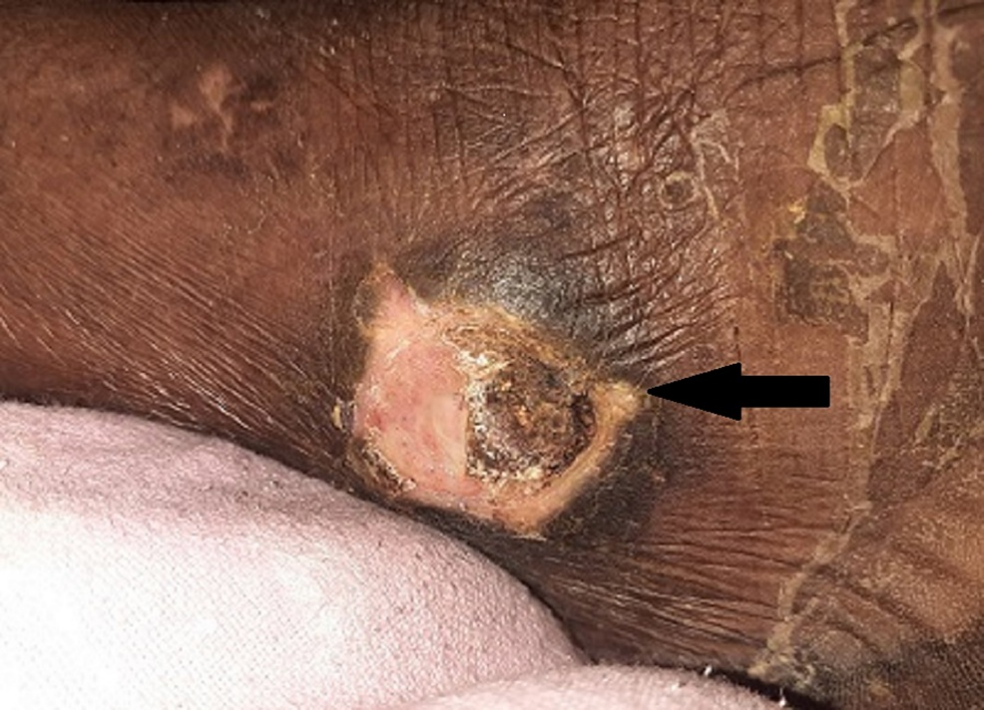
Image showing healing wound in right lower limb

A 57-year-old diabetic male came to our institute with complaints of right lower limb pain and swelling, along with high-grade fever for two weeks and shortness of breath for two days. Chest X-ray showed bilateral patchy consolidation of upper lobes (Figure 5), and the right lower limb had features of cellulitis (Figure 6). The patient was intubated and ventilated in view of severe respiratory distress. Blood cultures were sent, which grows B. pseudomallei sensitive to meropenem. Sputum examination was non-contributory.

The patient received parenteral antibiotics and other supportive measures in ICU and was extubated on day eight. The patient received 25 days of parenteral meropenam therapy. The patient improved and was discharged on oral cotrimoxazole.

Case 6

A 43-year-old male, working as a scientist in a microbiology lab at a medical college with no previous significant comorbidities, came to our institute with complaints of high-grade fever for 10 days. The patient was worked up for tropical diseases (in view of short history) such as malaria, dengue, leptospira, and scrub typhus, which came out to be negative, and empirical antibiotics were started. The patient was also diagnosed with diabetes mellitus (during hospital stay), with HbA1c of 9.2%. A blood culture was sent, which grew B. pseudomallei. He was started on antibiotics as per sensitivity report. On the third day of admission, the patient develops synovitis of the left ankle. After seven days of parenteral therapy, the patient improved, the swelling subsided along with the fever, and he was discharged on oral cotrimoxazole for three months.

Case 7

**Figure 7:**
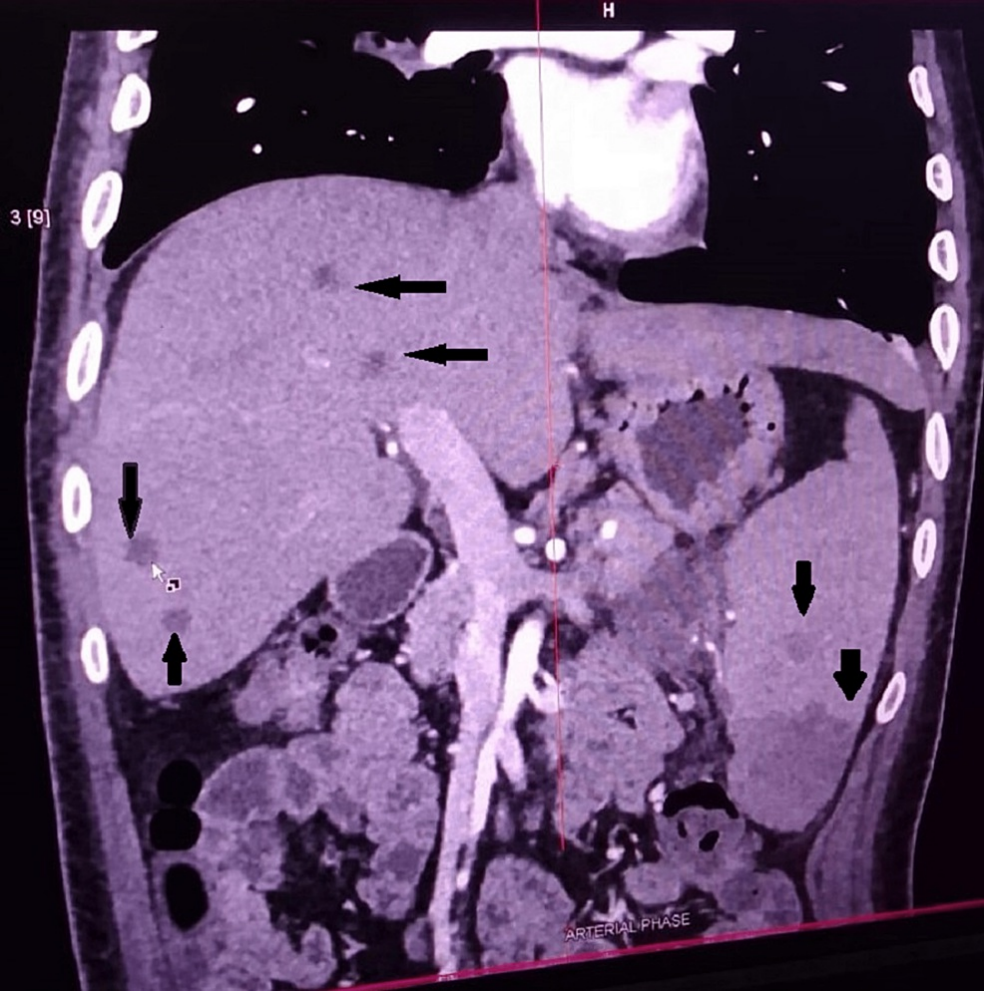
CT abdomen image showing multiple liver abscesses, splenic abscesses, and splenic infarcts in the coronal section

A 32-year-old male, diabetic and chronic alcoholic, came with complaints of pain in the abdomen and fever for 15 days. On examination, there was tenderness in the right hypochondrium. On evaluation, ultrasonography revealed multiple hypoechoic lesions in segment VI of the liver likely abscess, one of which was aspirated and sent for culture. CECT of the chest and abdomen also revealed multiple abscesses in the liver and multiple infarcts and abscess in the spleen (Figure 7), along with cavitary consolidation of the right middle lobe of the lung, with bilateral pleural effusion. Culture of aspirated pus grew B. pseudomallei sensitive to meropenem. The patient started on meropenem and improved over the course of 10 days. The patient was dischaged on oral cotrimoxazole for the next three months.

## Discussion

Melioidosis infection is more common than we suspect in regions other than where it is more prevalent. This is owing to a lack of suspicion, lack of medical facilities, and lack of understanding among health professionals regarding the infection over the years, since it also mimics the patient profile of other diseases with similar symptomatology. This is why it is a less suspected, underdiagnosed, and under-reported condition. Melioidosis presents a wide range of symptoms, ranging from asymptomatic infection to local abscesses, lower respiratory tract infection, and disseminated disease [5], and we found these variations in the seven cases described above.

Things have changed over the last few years as many cases of melioidosis have been early diagnosed, treated, and reported, which is a result of increasing awareness among health professionals, increased suspicion, and improved medical facilities in the form of availability, rapid results and accuracy of diagnostic tests, and advances in referral systems and treatment modality. Our purpose in reporting this case series of melioidosis is to increase general awareness about melioidosis among health professionals.

Diabetes and melioidosis were shown to be associated in 76% of cases in a study by Vidyalakshmi et al. [12]. Since diabetes mellitus has been identified as a significant risk factor, it has been hypothesized that rising diabetes rates are most likely to blame for rising melioidosis rates [13]. Alhatmi et al. [14] previously reported a case series of two cases, in which one patient lived, while the other could not be saved despite receiving the best ICU support and antibiotic treatment. Similar to this, Deshmukh et al. [15] described a case of a 43-year-old man who passed away from melioidosis as a result of the patient's inability to receive the proper antibiotics due to a delay in diagnosis. A comparable incident involving a 10-year-old boy who was unable to recover from septicemia because of a delayed diagnosis was reported by Miralles et al. [16].

Numerous recorded instances were from the southern region of India; however, this is not true of central India, where just a small number of cases were documented. We are, thus, documenting incidences of melioidosis in central India through this case series, which is pretty important as it will aid in raising awareness about this mystique disease.

All our melioidosis cases were diabetic, suggesting that diabetes is a significant risk factor for melioidosis. Joint involvement was present among all cases under study, which is a known but rare entity; similar joint involvement was seen in the study conducted by Raja et al. in 2016 [10]. B. pseudomallei is sensitive to carbapenem in all study cases although it may or may not be sensitive to other antibiotics tested, which indicates that B. pseudomallei strains are generally sensitive to carbapenem and that carbapenem-resistant strains of B. pseudomallei are rare [17,18].

## Conclusions

As a result, we draw the conclusion that melioidosis is more common than we realize in many locations. As a result, medical personnel should consider ruling out B. pseudomallei (melioidosis), in addition to other causes, when treating patients who exhibit fever and multiple abscesses in various organs. All B. pseudomallei strains found in our patients were sensitive to carbapenems, despite reports of many B. pseudomallei strains being resistant to common antibiotics. Populations with diabetes mellitus are more likely to develop melioidosis. Joint involvement in melioidosis is an established entity as either a direct extension of infection or a part of the disease's systemic response. I would suggest that all abscesses and big joint collections, if possible, must be drained, and pus should be sent for bacteriological confirmation, and culture and sensitivity report should be obtained.
